# Supplementary material for: Perceptions and acceptability of microsampling in children and young people: a single-centre survey
Source: BMJ Paediatr Open. 2022 Dec 30;6(1):e001716. doi: 10.1136/bmjpo-2022-001716 (PMC9809289; doi:10.1136/bmjpo-2022-001716)
Supplement: Supplementary data [file bmjpo-2022-001716supp001.pdf]

Supplementary Information

How are micro-sampling and home testing kits perceived by children and young people?

Andrew J Chetwynd[1,2], Julien Marro[1], Laura Whitty[1], Jenny Ainsworth[3], Jennifer Preston[1], Alan Salama[4], Louise Oni[1,5]

[1] Department of Women's and Children's Health, Institute of Life Course and Medical Sciences, University of Liverpool, Liverpool L12 2AP, UK

[2] Centre for Proteome Research, Institute of Systems, Molecular and Integrative Biology, University of Liverpool, Liverpool, U.K.

[3]NIHR Alder Hey CRF, Institute in the Park, Alder Hey Children's NHS Foundation Trust, Eaton Rd, Liverpool, L12 2AP

[4] Department of Renal Medicine, University College London, London, United Kingdom

[5] Department of Paediatric Nephrology, Alder Hey Children's NHS Foundation Trust Hospital, Liverpool L12 2AP, UK

Page 2: Questionnaire used during PPIE event

Page 3: Compiled responses to questionnaire in table format

Questionnaire for PPIE event held on 7th April 2022

Do you think young people with inflammatory diseases would prefer this method compared to visiting Doctors or hospital?

Yes/No/Unsure

Do you think young people with inflammatory diseases would be more likely to participate in a clinical research study using this method?

Yes/No/Unsure

Do you think young people with inflammatory diseases would be willing to use these devices to provide urine samples?

Yes/No//Unsure

How frequently do you think young people with inflammatory diseases would be happy to provide a urine sample?
Weekly/every 2 weeks/monthly/every 3 months/less often/Unsure

Do you think young people with inflammatory diseases would be willing to use these devices to provide blood samples?

Yes/No/Unsure

How frequently do you think young people with inflammatory diseases would be happy to provide a blood sample?
Weekly/every 2 weeks/monthly/every 3 months/less often/Unsure

Did the pack fit through your letter box?

Yes/No/Unsure

Table 1: Compiled results of questionnaire

Question	Yes	No	Unsure	Once a week	Every 2 weeks	Once a month	Once every 3 months	Less often
1	9	0	1	N/A	N/A	N/A	N/A	N/A
2	8	0	2	N/A	N/A	N/A	N/A	N/A
3	8	0	2	N/A	N/A	N/A	N/A	N/A
4	N/A	N/A	0	4	2	4	0	0
5	9	0	1	N/A	N/A	N/A	N/A	N/A
6	N/A	N/A	0	2	3	5	0	0
7*	1	0	0	N/A	N/A	N/A	N/A	N/A

*n=1 as only one participant who was able to attend had received a pack, another participant who had been shipped a pack had yet to receive it.
