# Supplementary material for: Perceptions and acceptability of microsampling in children and young people: a single-centre survey
Source: BMJ Paediatr Open. 2022 Dec 30;6(1):e001716. doi: 10.1136/bmjpo-2022-001716 (PMC9809289; doi:10.1136/bmjpo-2022-001716)
Supplement: Author's manuscript [file bmjpo-2022-001716.draft_revisions.pdf]

BMJ Paediatrics Open

BMJ Paediatrics Open is committed to open peer review. As part of this commitment we make the peer review history of every article we publish publicly available.

When an article is published we post the peer reviewers' comments and the authors' responses online. We also post the versions of the paper that were used during peer review. These are the versions that the peer review comments apply to.

The versions of the paper that follow are the versions that were submitted during the peer review process. They are not the versions of record or the final published versions. They should not be cited or distributed as the published version of this manuscript.

BMJ Paediatrics Open is an open access journal and the full, final, typeset and author-corrected version of record of the manuscript is available on our site with no access controls, subscription charges or pay-per-view fees (http://bmjpaedsopen.bmj.com).

If you have any questions on BMJ Paediatrics Open's open peer review process please email info.bmjpo@bmj.com

BMJ Paediatrics Open

How are micro-sampling and home testing kits perceived by children and young people?

Journal:	*BMJ Paediatrics Open*
Manuscript ID	bmjpo-2022-001716
Article Type:	Original research
Date Submitted by the Author:	17-Oct-2022
Complete List of Authors:	Chetwynd, Andrew; University of Liverpool, Women`s and Children`s Health<br>Marro, Julien; University of Liverpool, Department of Women's and Children's Health, Institute in the Park<br>Whitty, Laura; University of Liverpool, Department of Women's and Children's Health, Institute in the Park<br>Ainsworth, Jenny; NIHR Alder Hey CRF, Institute in the Park, Alder Hey Children`s NHS Foundation Trust<br>Preston, Jennifer; NIHR Alder Hey CRF, Institute in the Park, Alder Hey Children`s NHS Foundation Trust<br>Salama, Alan; University College London, Department of Renal Medicine<br>Oni, Louise; University of Liverpool, Department of Women's and Children's Health; Alder Hey Children's NHS Foundation Trust Hospital, Department of Paediatric Nephrology
Keywords:	Ethics, Qualitative research, Technology, Adolescent Health, Nephrology

SCHOLARONE™
Manuscripts

I, the Submitting Author has the right to grant and does grant on behalf of all authors of the Work (as defined in the below author licence), an exclusive licence and/or a non-exclusive licence for contributions from authors who are: i) UK Crown employees; ii) where BMJ has agreed a CC-BY licence shall apply, and/or iii) in accordance with the terms applicable for US Federal Government officers or employees acting as part of their official duties; on a worldwide, perpetual, irrevocable, royalty-free basis to BMJ Publishing Group Ltd ("BMJ") its licensees and where the relevant Journal is co-owned by BMJ to the co-owners of the Journal, to publish the Work in this journal and any other BMJ products and to exploit all rights, as set out in our licence.

The Submitting Author accepts and understands that any supply made under these terms is made by BMJ to the Submitting Author unless you are acting as an employee on behalf of your employer or a postgraduate student of an affiliated institution which is paying any applicable article publishing charge ("APC") for Open Access articles. Where the Submitting Author wishes to make the Work available on an Open Access basis (and intends to pay the relevant APC), the terms of reuse of such Open Access shall be governed by a Creative Commons licence – details of these licences and which Creative Commons licence will apply to this Work are set out in our licence referred to above.

Other than as permitted in any relevant BMJ Author's Self Archiving Policies, I confirm this Work has not been accepted for publication elsewhere, is not being considered for publication elsewhere and does not duplicate material already published. I confirm all authors consent to publication of this Work and authorise the granting of this licence.

Confidential: For Review Only

How are micro-sampling and home testing kits perceived by children and young people?

Andrew J Chetwynd[1,2], Julien Marro[1], Laura Whitty[1], Jenny Ainsworth[3], Jennifer Preston[1,3], Alan Salama[4], Louise Oni[1,5*]

[1] Department of Women's and Children's Health, Institute of Life Course and Medical Sciences, University of Liverpool, Liverpool L12 2AP, UK

[2] Centre for Proteome Research, Institute of Systems, Molecular and Integrative Biology, University of Liverpool, Liverpool, U.K.

[3]NIHR Alder Hey CRF, Institute in the Park, Alder Hey Children's NHS Foundation Trust, Eaton Rd, Liverpool, L12 2AP

[4] Department of Renal Medicine, University College London, London, United Kingdom

[5] Department of Paediatric Nephrology, Alder Hey Children's NHS Foundation Trust Hospital, Liverpool L12 2AP, UK

***To whom correspondence should be addressed**

louise.oni@liverpool.ac.uk

Tel.: +44-(0)151-252-54

Keywords

Children, VAMS, DBS, Dried blood spot, Dried urine spot, consent, clinical science

Funding

This research was funded by the Funding Autoimmune Research (F.A.I.R.) Charity (Registered U.K. Charity; number: 1176388) and Kidney Research UK Meditech academy programme The Liverpool GenR YPAG is funded by the NIHR Alder Hey Children`s NHS Foundation Trust Clinical Research Facility (CRF). This work was carried outwith in the Experimental Arthritis Treatment centre for children funded by Versus Arthritis.

https://mc.manuscriptcentral.com/bmjpo

Author contributions

LO and AS obtained funding and led the concepts behind aspects of the PPI event. LO, AJC, JM, LW and JA produced the demo kits and posted out to participants. AJC and LO wrote the initial draft of the manuscript. JM kept minutes of the PPI event. LW, JP and JA arranged and set up the PPI event and JA led the overall PPI session with LO and AJC presenting the sampling technique and providing the research background. JP manages the PPIE programme and has contributed to the final manuscript. All authors were responsible for critical revision of the manuscript and approved the final version.

Ethical and regulatory approval

All work with GenR Liverpool was carried out after detailed meetings and approved via a pre-defined Researcher Agreement with GenR members and authorised by the PPIE Manager (2022).

Conflict of Interest

The authors have no conflicts of interest to report in this work.

Data Availability

All data relevant to this work are presented in the article or in the associated supplemental information.

What is known about the subject?

- New born screening using dried blood spots have been used for decades and the use of dried blood spots is adults is growing in popularity.
- The use of dried blood spots in adults has been shown to be applicable for many questions such as adherence to treatment regime.
- There is evidence from adults that they prefer this method of sample collection due to its less invasive nature particularly when economic and time costs are accounted for.

What this study adds?

- We carried out a study to determine the views and perceptions of a small group of children and young persons on the collection of dried blood spots.
- We show for the first time that this age group are happy to provide samples in this manner and the frequency of sampling that they may find acceptable.
- This opens the possibility for more children and young persons to contribute to medical research whom may have been excluded previously and may ease longitudinal sampling.

Confidential: For Review Only

Abstract

Background

The use of at home micro-sampling devices, such as dried aliquots of blood and urine, for scientific research has expanded in recent years. These devices collect small volumes of biofluids which air dry and can be posted to central laboratories. In general, they are cheaper and more convenient, saving patients travel time and freeing up staff. In adult populations, this sampling method is well perceived, with >90% of samples being of adequate size and quality for scientific research. However, little literature on micro-sampling in paediatric populations exists. The aim of this study was to explore the perceptions and acceptability of micro-sampling in children and young people.

Methods

Online Patient and Public Involvement was obtained by attending the Generation R Young Person's Advisory Group Liverpool, to explore the perceptions of at home micro-sampling. During the session, the rationale and use of micro-sampling was demonstrated. The participants provided informal feedback during the interactive session and completed a short online questionnaire.

Results

A total of 13 children and young people attended the event and they were aged 10-17 years. The general feedback on micro-sampling was positive with ≥80% of respondents indicating they would be willing to participate in at home micro-sampling studies. Furthermore, 100% of respondants reported being willing to provide both biofluids on a monthly basis and 40% would be willing to provide dried urine samples weekly.

Conclusions

Children and young people are supportive of at home micro-sampling for research purposes and this offers the possibility of widening participation to research.

https://mc.manuscriptcentral.com/bmjpo

Confidential: For Review Only

Background

The collection of biofluid samples from patients for basic science and clinical research typically requires a patient to attend an appointment with a medical practitioner, particularly in the case of providing blood samples[1,2]. Even in the case of a urine sample, a visit to a hospital or research facility is frequently required, incuring further costs and time commitments to families. Recently, there has been a growing interest in micro sampling such as dried blood and urine spots, collected at home and posted back to the research centre for analysis[2]. The use of dried biofluids is cheaper[1,3,4] and an array of studies show the samples are stable over a few days which encompass the necessary postal time[5]. In addition, as a dried biofluid, the biological risk associated with the liquid form is significantly reduced[2,6]. Since the Covid19 pandemic, home testing of nasal/throat swabs have become common practice.

To date, there has been a growing number of studies evaluating home sampling and postage delivery spanning personalised nutrition[8], genome testing[9], disease monitoring and pre-clinical research[4,10,11]. In addition to the scientific questions at the heart of these studies, a number have looked into patients' perception and sample quality of micro-sampling. The overarching consensus is that participants are happy to provide blood, urine and saliva samples using micro sampling devices with <10% of patients preferring traditional clinical visits. The reasons for the latter being due to confusion over the sample collection, fear of pain with the finger prick blood devices or not feeling adequately trained in the sampling technique[12]. Furthermore, these studies report high levels of compliance with >90% of expected samples being taken and successfully arriving for analysis, even in the case of longitudinal sampling[3,13].

These studies highlight the promise of at home micro-sampling to increase recruitment of patient populations that are not encompassed within a research-active hospital or university region and facilitate larger cohort recruitment with longitudinal sampling studies[4].

Confidential: For Review Only

There is little data evaluating at home micro-sampling devices in paediatric cohorts, despite paediatric healthcare professionals being familiar with national newborn screening programmes. Using this technique in children and young people may also be dependent on the willingness of research ethics committees to grant ethical approval for at-home micro-sampling testing in a vulnerable population and thus there is a threat that children may be excluded from this research area. The aim of this work is to explore the perceptions of and willingness of children and young people to partake in research focused on at-home micro-sampling of biofluids.

Methods

Researchers (L.O (Female), A.J.C (Male)) applied to attend one of the GenR YPAG Liverpool meetings. The Liverpool YPAG was set up in 2006 and is open to young people between the ages of 8-23 years. The group currently has 28 members aged between 10-22 years old. Members either have experience of taking part in health research, have experience of living with a condition or disability, or have a general interest in learning about medicine/research/science. There were no inclusion or exclusion requirements for the study other than being a member of GenR YPAG group, any member was free to participate.

Prior to the workshop, meetings were held with the group facilitator (J.A) to plan the session as part of a regular GenR YPAG meeting and to support the purpose of the activity including expected outputs. The dissemination of a sample of the pack were also arranged. It was decided to send packs to a small group of members who would demonstate to the rest of the group during the meeting. In total 6 mock sampling packs were sent to YPAG members to facilitate a live demonstration. In the mock sampling pack, volumetric absorptive micro-sampling (VAMS, Neoteryx, CA, U.S.A) devices were provided with 2 aliquots of food colouring to mimic blood and urine. Two aliquots of water were also provided to allow a demonstration of how to recover the dried simulated biofluids.

The online meeting was conducted over 45 minutes using Zoom video conferencing software (Zoom video communications, Ca, U.S.A), the session was not recorded. Researchers (L.O, J.A) presented

https://mc.manuscriptcentral.com/bmjpo

current recruitment and sample collection practices and the importance of these for understanding inflammatory renal disease. The session subsequently involved a short video and a live interactive demonstration (A.J.C) to illustrate the sample collection process using the mock sampling kits. Mock samples (VAMS and food colouring) were left to dry for 20 minutes during the discussion phase prior to illustrating how to recover the dried sample using the provided water.

During the presentation a short video was shown demonstrating the use of a finger prick device for the generation of a capillary whole blood sample and its collection with a VAMS device (Figure 1). The group then discussed with the whole group for 35 minutes the positive and negative points to sample collection and finally a multiplechoice questionnaire (SurveyMonkey Audience www.surveymonkey.com/mp/audience) (Supplementary Information 1) was circulated to assess their perception and willingness for children and young people to partake in studies using micro-sampling devices. During the meeting written minutes were recorded by 2 individuals (L.O and J.M). The responces to the multiplechoice questionnaire was tallied and graphs produced (A.J.C) using GraphPad Prism Version 8.1.1 (GraphPad Software, CA, USA). The trends in perception were then garnered from this compiled data.

Confidential: For Review Only

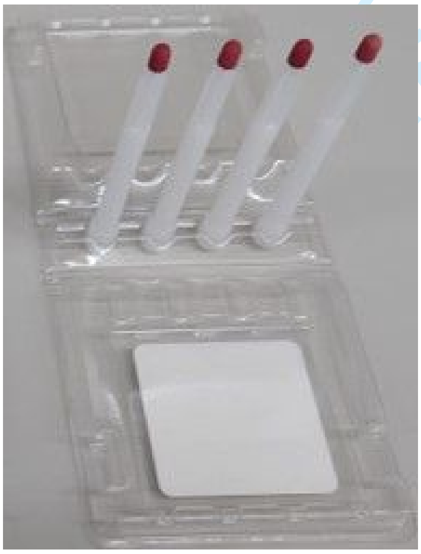

Figure 1: VAMS devices for the collection dried blood spots generated from 30 µL of capillary blood

Patient/public involvement

This work collected the views and perceptions of children and young persons from the public to inform and improve future at home dried biofluid micro-sampling clinical research studies.

Results

Participants

In total, 13 young people (46% of members, no parents present) attended the YPAG meeting via Zoom from home in April 2022. Only 1 young person of 6 received the mock pack and was able to attend due to educational and personal commitments. They confirmed that the mock sampling pack was able to fit through their letter box and had been received intact. Due to the small group data saturation was not reached.

Findings from informal discussion

When asked for first impressions on micro-sampling, the feedback was positive with comments on the method being "quick", "efficient", "simple", "helpful", "fast", "great" and "easy" being made. The participants were asked for any advantages of using these micro-sampling collection kits which followed a similar trend with responses of "Don't need a lot of volume of urine/blood", "simple to use and also fewer travelling costs" and "only takes ten minutes rather than a day to go to hospital".

Conversely, a number of concerns were raised which can either be readily resolved with added information or require additional work prior to rolling out micro-sampling (Table 1). The participant doing the hands-on demonstration mentioned that initially the procedure seemed "complicated" but ended up being "easy and simple to operate". This raised a further question "will there be an instruction guide or video?", the initial idea behind this form of sample collection was to provide a manual with a step by step process. However, the group suggested using a QR code with an embedded link to a guidance video or online manual.

Confidential: For Review Only
https://mc.manuscriptcentral.com/bmjpo

An issue was raised about whether the results from these samples are comparable to standard onsite venous blood and urine sample collection. The issue of still having to collect a urine sample in a container prior to taking the microsample was also raised highlighting the need to investigate an appropriate container in the future. However, it was emphasised that collecting a urine sample at home was more convenient and more private compared to a hospital visit. The supply of finger prick devices in the packs to be shipped out was also questioned and it was confirmed that plans would be to include these with spares in case one did not work or multiple pricks were required. The final concern raised was the environmental impact of such testing, demonstrating the environmental concern of patients alongside medical concerns.

Table 1: Concerns raised by the group and the impact they may have on future studies with potential solutions discussed

**Concerns raised**	**Potential issues this would have**	**Possible solutions**
Complexity of sample collection/requirement for instruction	Poor quality sample<br>Unnecessary pain if blood taken incorrectly<br>Stress of participant	Develop manuals and videos in accessible language<br>Use of further PPI events to evaluate instructional manuals and videos.<br>Incorporation of QR codes to link to videos and manuals
Lack of data on how micro-sampling compared to traditional techniques	Lower quality data being collected<br>Not enough samples<br>Sample degradation	Prior to each study a method development phase is required to optimise sample recovery, limits of detection, sample stability.

Confidential: For Review Only

Urine collection in a separate container	Inconvenient<br>Large volume to dispose of without spilling	Focus group highlighted that this is still preferable to in person sampling in clinic. However, it's something to consider for researchers to the process as easy as possible.
Will the kits posted home contain the finger prick devices?	It could limit the recruitment of patients if they think they need to provide materials for sample collection	Full description of the process and kit contents in information packs for participants.<br>Ensuring adequate information in manuals and instructional videos.
Environmental impact of the micro-sampling kits	If perceived to be greater than traditional approaches it may deter participants.<br>It's important that clinical researchers consider the impact upon global pollution burden as this contributes to poor health outcomes.	At home sample collection reduces the need for transport to a research site.<br>Less plastics are required compared to traditional methods.<br>Researchers should assess a number of micro-sampling devices to minimise the use of single use plastics.

1

Confidential: For Review Only

In terms of the ages that the group thought would be appropriate for micro-sampling, there was no obvious age cut-off. They suggested that it would need to be done on a case by case basis and felt that if young people under 16 years old were able to understand and consent then they should be allowed to participate and that some children and young people may actually prefer this method of sampling compared to traditional venous sample collection.

Findings from questionnaire

The YPAG were asked to answer a multiple choice questionnaire (Supplementary information and Supplementary Table 1) to help evaluate their perceptions. Initial questions revolved around the use of micro-sampling devices for clinical research in paediatric cohorts. In total, 10 participants (77%) provided feedback to the questionnaire and of those, 90% felt that micro-sampling was preferential compared to having to visit the GP or a hospital site for blood drawing and 10% of respondents were unsure. Furthermore, 80% of respondents thought that this approach would increase the likelihood of patients wishing to participate in clinical research while 20% were unsure.

A key question with this type of micro-sampling in this population is to understand which biofluids they feel comfortable with providing and this may provide challenging for ethical committees to make an informed decision on their behalf. Perhaps surprisingly, a great proportion were willing to provide a blood sample (90%) compared to a urine sample (80%), despite the use of a finger prick test to obtain the capillary blood sample. In the questions regarding both the perception of the micro-sampling devices and the biofluids, between 10 and 20% of respondents replied with 'unsure'.

Confidential: For Review Only

Finally, the frequency of sampling is significant for the recruitment of patients over a defined time frame with periodic sampling providing a great opportunity for longitudinal data collection as the disease changes over time and treatment takes effect. Perhaps unsurprisingly, there was a trend for blood to be collected on a less regular basis, potentially due to apprehension regarding the finger prick testing. The frequency of urine testing was less clear cut with 40% suggesting that weekly collection or monthly collection would be acceptable (Figure 2).

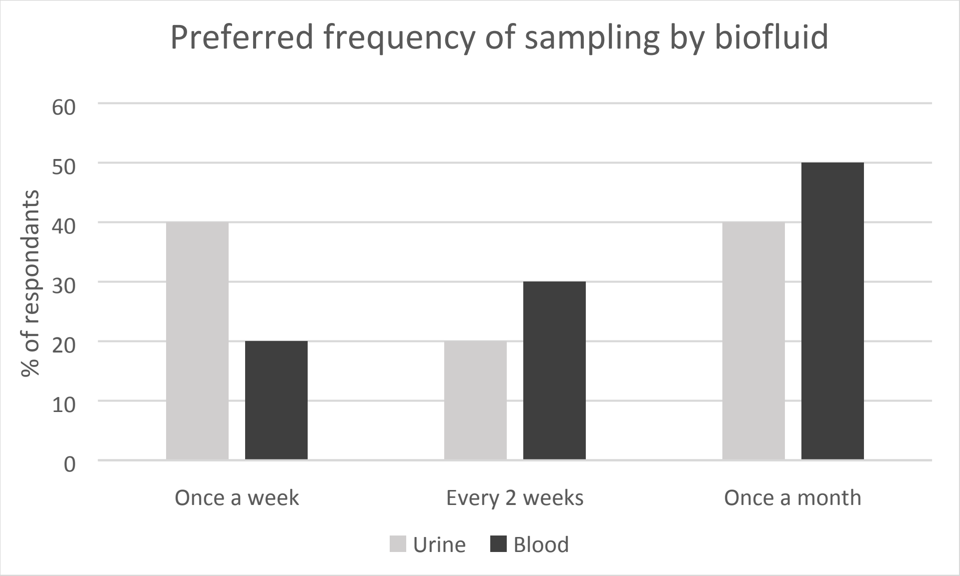

Figure 2: The Liverpool YPAG opinions on the hypothetical acceptable frequency of micro-sampling depending upon sample type.

Discussion

Initial discussion

The initial positive responses to the use of at home micro-sampling and return by mail reflect the findings in adult cohorts about the use of such devices that they would be quicker and easier to use and cut down on time and economic costs associated with a hospital or GP visit[13].

Importantly, a number of potential weaknesses were also highlighted by the group. The issues raised over the complexity and level of instruction for the at home sampling is a vital aspect of improving inclusivity enabling children, young people and parents to make informed choices and safely and

reliably carry out the sample collection procedure. In order to be as inclusive as possible with this style of sampling, there is a clear need for providing instructions accessible for all to ensure safe and reliable at home micro-sample collection. A further PPI event would be beneficial to evaluate the manual or videos in order to optimise these for this audience. These issues are also reflected in adult cohorts where a number of participants requested further guidance for micro-sampling and that when it was made available participation rates were increased[3,12].

Concerns regarding quality of data that can be ascertained from micro-sampling compared to more traditional approaches is an understandable concern as patients may be asked to provide samples more frequently than typical during their clinical follow-up so it is important that researchers can reassure patients that the samples they are providing are being put to good use. In an array of studies in adult populations >90% of returned samples were of adequate volume and quality for research purposes, suggesting that this approach would be appropriate[3,12–14]. However, validation and stability for the specific analytes of interest will be required for all studies using this form of sample collection, particularly given that reference ranges are typically provided for serum or plasma rather than whole blood[12,15]. Once this has been validated, it would possibly be useful to include this information and additional information of previously published studies to show the applicability of the sampling technique to potential patients interested in providing dried biofluid samples.

The group highlighted a concern of the environmental impact of the plastics usage with this micro-sampling technique. Investigators will need to consider the type of plastic and that by removing the requirement to travel to a research centre and the reduced sample processing in the lab[1,3] may balance the environmental impact compared to conventional sampling. Furthermore, the suggestion of QR codes for access to videos and manuals could reduce paper work and the weight of the packaging however this may skew against individuals in lower socio-economic backgrounds without access to take advantage of this.

PPI Questionnaire

https://mc.manuscriptcentral.com/bmjpo

Confidential: For Review Only

The reported willingness to participate in this style of study is much higher than the response rate observed in an adult epidemiological cohort and may reflect that this was a small group or the difference between a theoretical study and one that is actually taking place[14]. This is key as this population is already under represented in the literature potentially due to the low numbers of specialist paediatric research facilities, spare biofluid volume for clinical research or reticence to provide ethical consent for clinical research on this population. All of which combine to limit the amount of research on paediatric diseases potentially leaving this population behind in terms of mechanistic insights, discovery of potential drug targets and the development of new drugs.

The slight preference for blood sampling over urine is particularly interesting because urine is often stated as a preferred biofluid for research due to it being less invasive and more plentiful. It is possible that this is down to having to provide a sample into a large pot and then taking a sample or embarrassment around providing a urine sample. In addition, the disposal of a container full of urine once the sample has been taken may also put participants off.

The YPAG group responded that they would be open to longitudinal collection of biofluids, though there was no consensus regarding the frequency of sample collection. The results suggested that aiming monthly sampling may be acceptable however further work using specific cohorts may be required.

Conclusions

The workshop demonstrated for the first time that children and young people are overall supportive of at home micro-sampling for clinical research. While the group size is small and there are biases due to the intellect and interest in clinical research held by members possibly skewing the findings, we are able to report that children and young people may be willing to provide samples in this manner. We also gained insight on the frequency and additional concerns that need to be addressed for longitudinal research studies aimed at elucidating mechanistic insights into paediatric diseases. This work is the first to collect thoughts and perspectives of children and young persons on at home micro-

https://mc.manuscriptcentral.com/bmjpo

Confidential: For Review Only

sampling for multiple biofluids. As such it may help applications to ethics boards for this style of research now that there are limited data on the views of this age group.

Acknowledgements

We would like to thank the Liverpool GenR YPAG for allowing us to present our proposal and for their critical and thoughtful feedback to this work.

References

1 Locatelli M, Tartaglia A, D'Ambrosio F, *et al.* Biofluid sampler: A new gateway for mail-in-analysis of whole blood samples. *Journal of Chromatography B* 2020;**1143**:122055. doi:10.1016/J.JCHROMB.2020.122055

2 Resano M, Belarra MA, García-Ruiz E, *et al.* Dried matrix spots and clinical elemental analysis. Current status, difficulties, and opportunities. *TrAC Trends in Analytical Chemistry* 2018;**99**:75–87. doi:10.1016/J.TRAC.2017.12.004

3 Roberts SC, Seav SM, McDade TW, *et al.* Self-collected dried blood spots as a tool for measuring ovarian reserve in young female cancer survivors. *Human Reproduction* 2016;**31**:1570–8. doi:10.1093/HUMREP/DEW114

4 Parker SP, Cubitt WD. The use of the dried blood spot sample in epidemiological studies. *J Clin Pathol* 1999;**52**:633–9.

5 Nakajima D, Ohara O, Kawashima Y. Toward proteome-wide exploration of proteins in dried blood spots using liquid chromatography-coupled mass spectrometry. *Proteomics* 2021;**21**:2100019. doi:10.1002/PMIC.202100019

6 Schmidt J, Lindemann V, Olsen M, *et al.* Dried urine spots as sampling technique for multi-mycotoxin analysis in human urine. *Mycotoxin Res* 2021;**37**:129–40. doi:10.1007/S12550-021-00423-1/TABLES/3

7 Protti M, Mandrioli R, Mercolini L. Quantitative microsampling for bioanalytical applications related to the SARS-CoV-2 pandemic: Usefulness, benefits and pitfalls. *J Pharm Biomed Anal* 2020;**191**:113597. doi:10.1016/J.JPBA.2020.113597

8 Hoeller U, Baur M, Roos FF, *et al.* Application of dried blood spots to determine vitamin D status in a large nutritional study with unsupervised sampling: the Food4Me project. *British Journal of Nutrition* 2016;**115**:202–11. doi:10.1017/S0007114515004298

9 Agrawal P, Katragadda S, Hariharan AK, *et al.* Validation of whole genome sequencing from dried blood spots. *BMC Med Genomics* 2021;**14**:1–11. doi:10.1186/S12920-021-00951-W/FIGURES/2

10 Solomon SS, Solomon S, Rodriguez II, *et al.* Dried blood spots (DBS): A valuable tool for HIV surveillance in developing/tropical countries. *Int J STD AIDS* 2002;**13**:25–8. doi:10.1258/0956462021924578

Confidential: For Review Only

11 Chace DH, Millington DS, Terada N, *et al.* Rapid diagnosis of phenylketonuria by quantitative analysis for phenylalanine and tyrosine in neonatal blood spots by tandem mass spectrometry. *Clin Chem* 1993;**39**:66–71. doi:10.1093/CLINCHEM/39.1.66

12 Williams SR, McDade TW. The Use of Dried Blood Spot Sampling in the National Social Life, Health, and Aging Project. *The Journals of Gerontology: Series B* 2009;**64B**:i131–6. doi:10.1093/GERONB/GBN022

13 Spielberg F, Critchlow C, Vittinghoff E, *et al.* Home collection for frequent HIV testing: acceptability of oral ¯uids, dried blood spots and telephone results. *Lippincott Williams & Wilkins AIDS* 2000;**14**:1819–28.http://journals.lww.com/aidsonline (accessed 31 Mar 2022).

14 Sakhi AK, Bastani NE, Ellingjord-Dale M, *et al.* Feasibility of self-sampled dried blood spot and saliva samples sent by mail in a population-based study. *BMC Cancer* 2015;**15**:1–9. doi:10.1186/S12885-015-1275-0/TABLES/3

15 McClendon-Weary B, Putnick DL, Robinson S, *et al.* Little to Give, Much to Gain—What Can You Do With a Dried Blood Spot? *Curr Environ Health Rep* 2020;**7**:211–21. doi:10.1007/S40572-020-00289-Y/FIGURES/1

Confidential: For Review Only

Supplementary Information

How are micro-sampling and home testing kits perceived by children and young people?

Andrew J Chetwynd[1,2], Julien Marro[1], Laura Whitty[1], Jenny Ainsworth[3], Jennifer Preston[1], Alan Salama[4], Louise Oni[1,5]

[1] Department of Women's and Children's Health, Institute of Life Course and Medical Sciences, University of Liverpool, Liverpool L12 2AP, UK

[2] Centre for Proteome Research, Institute of Systems, Molecular and Integrative Biology, University of Liverpool, Liverpool, U.K.

[3]NIHR Alder Hey CRF, Institute in the Park, Alder Hey Children's NHS Foundation Trust, Eaton Rd, Liverpool, L12 2AP

[4] Department of Renal Medicine, University College London, London, United Kingdom

[5] Department of Paediatric Nephrology, Alder Hey Children's NHS Foundation Trust Hospital, Liverpool L12 2AP, UK

Page 2: Questionnaire used during PPIE event

Page 3: Compiled responses to questionnaire in table format

Confidential: For Review Only

Questionnaire for PPIE event held on 7th April 2022

Do you think young people with inflammatory diseases would prefer this method compared to visiting Doctors or hospital?

Yes/No/Unsure

Do you think young people with inflammatory diseases would be more likely to participate in a clinical research study using this method?

Yes/No/Unsure

Do you think young people with inflammatory diseases would be willing to use these devices to provide urine samples?

Yes/No//Unsure

How frequently do you think young people with inflammatory diseases would be happy to provide a urine sample?
Weekly/every 2 weeks/monthly/every 3 months/less often/Unsure

Do you think young people with inflammatory diseases would be willing to use these devices to provide blood samples?

Yes/No/Unsure

How frequently do you think young people with inflammatory diseases would be happy to provide a blood sample?
Weekly/every 2 weeks/monthly/every 3 months/less often/Unsure

Did the pack fit through your letter box?

Yes/No/Unsure

Confidential: For Review Only

Table 1: Compiled results of questionnaire

Question	Yes	No	Unsure	Once a week	Every 2 weeks	Once a month	Once every 3 months	Less often
1	9	0	1	N/A	N/A	N/A	N/A	N/A
2	8	0	2	N/A	N/A	N/A	N/A	N/A
3	8	0	2	N/A	N/A	N/A	N/A	N/A
4	N/A	N/A	0	4	2	4	0	0
5	9	0	1	N/A	N/A	N/A	N/A	N/A
6	N/A	N/A	0	2	3	5	0	0
7*	1	0	0	N/A	N/A	N/A	N/A	N/A

*n=1 as only one participant who was able to attend had received a pack, another participant who had been shipped a pack had yet to receive it.

Confidential: For Review Only

BMJ Paediatrics Open

Perceptions and acceptability of micro-sampling in children and young people; a single centre survey

Journal:	*BMJ Paediatrics Open*
Manuscript ID	bmjpo-2022-001716.R1
Article Type:	Original research
Date Submitted by the Author:	07-Dec-2022
Complete List of Authors:	Chetwynd, Andrew; University of Liverpool, Women`s and Children`s Health<br>Marro, Julien; University of Liverpool, Department of Women's and Children's Health, Institute in the Park<br>Whitty, Laura; University of Liverpool, Department of Women's and Children's Health, Institute in the Park<br>Ainsworth, Jenny; NIHR Alder Hey CRF, Institute in the Park, Alder Hey Children`s NHS Foundation Trust<br>Preston, Jennifer; NIHR Alder Hey CRF, Institute in the Park, Alder Hey Children`s NHS Foundation Trust<br>Salama, Alan; University College London, Department of Renal Medicine<br>Oni, Louise; University of Liverpool, Department of Women's and Children's Health; Alder Hey Children's NHS Foundation Trust Hospital, Department of Paediatric Nephrology
Keywords:	Ethics, Qualitative research, Technology, Adolescent Health, Nephrology

SCHOLARONE™
Manuscripts

I, the Submitting Author has the right to grant and does grant on behalf of all authors of the Work (as defined in the below author licence), an exclusive licence and/or a non-exclusive licence for contributions from authors who are: i) UK Crown employees; ii) where BMJ has agreed a CC-BY licence shall apply, and/or iii) in accordance with the terms applicable for US Federal Government officers or employees acting as part of their official duties; on a worldwide, perpetual, irrevocable, royalty-free basis to BMJ Publishing Group Ltd ("BMJ") its licensees and where the relevant Journal is co-owned by BMJ to the co-owners of the Journal, to publish the Work in this journal and any other BMJ products and to exploit all rights, as set out in our licence.

The Submitting Author accepts and understands that any supply made under these terms is made by BMJ to the Submitting Author unless you are acting as an employee on behalf of your employer or a postgraduate student of an affiliated institution which is paying any applicable article publishing charge ("APC") for Open Access articles. Where the Submitting Author wishes to make the Work available on an Open Access basis (and intends to pay the relevant APC), the terms of reuse of such Open Access shall be governed by a Creative Commons licence – details of these licences and which Creative Commons licence will apply to this Work are set out in our licence referred to above.

Other than as permitted in any relevant BMJ Author's Self Archiving Policies, I confirm this Work has not been accepted for publication elsewhere, is not being considered for publication elsewhere and does not duplicate material already published. I confirm all authors consent to publication of this Work and authorise the granting of this licence.

Confidential: For Review Only

Perceptions and acceptability of micro-sampling in children and young people; a single centre survey

Andrew J Chetwynd[1,2], Julien Marro[1], Laura Whitty[1], Jenny Ainsworth[3], Jennifer Preston[1,3], Alan Salama[4], Louise Oni[1,5*]

[1] Department of Women's and Children's Health, Institute of Life Course and Medical Sciences, University of Liverpool, Liverpool L12 2AP, UK

[2] Centre for Proteome Research, Institute of Systems, Molecular and Integrative Biology, University of Liverpool, Liverpool, U.K.

[3]NIHR Alder Hey CRF, Institute in the Park, Alder Hey Children's NHS Foundation Trust, Eaton Rd, Liverpool, L12 2AP

[4] Department of Renal Medicine, University College London, London, United Kingdom

[5] Department of Paediatric Nephrology, Alder Hey Children's NHS Foundation Trust Hospital, Liverpool L12 2AP, UK

***To whom correspondence should be addressed**

louise.oni@liverpool.ac.uk

Tel.: +44-(0)151-252-54

Keywords

Children, VAMS, DBS, Dried blood spot, Dried urine spot, consent, clinical science

Funding

This research was funded by the Funding Autoimmune Research (F.A.I.R.) Charity (Registered U.K. Charity; number: 1176388) and Kidney Research UK Meditech academy programme The Liverpool GenR YPAG is funded by the NIHR Alder Hey Children`s NHS Foundation Trust Clinical Research Facility (CRF). This work was carried out with in the Experimental Arthritis Treatment centre for children funded by Versus Arthritis.

https://mc.manuscriptcentral.com/bmjpo

Confidential: For Review Only

Author contributions

LO and AS obtained funding and led the concepts behind aspects of the PPI event. LO, AJC, JM, LW and JA produced the demo kits and posted out to participants. AJC and LO wrote the initial draft of the manuscript. JM kept minutes of the PPI event. LW, JP and JA arranged and set up the PPI event and JA led the overall PPI session with LO and AJC presenting the sampling technique and providing the research background. JP manages the PPIE programme and has contributed to the final manuscript. All authors were responsible for critical revision of the manuscript and approved the final version.

Ethical and regulatory approval

All work with GenR Liverpool was carried out after detailed meetings and approved via a pre-defined Researcher Agreement with GenR members and authorised by the PPIE Manager (2022).

Conflict of Interest

The authors have no conflicts of interest to report in this work.

Data Availability

All data relevant to this work are presented in the article or in the associated supplemental information.

Abstract

Background

The use of at home micro-sampling devices, such as dried aliquots of blood and urine, for scientific research has expanded in recent years. These devices collect small volumes of biofluids which air dry and can be posted to central laboratories. In general, they are cheaper and more convenient, saving patients travel time and freeing up staff. In adult populations, this sampling method is well perceived, with >90% of samples being of adequate size and quality for scientific research. However, little literature on micro-sampling in paediatric populations exists. The aim of this study was to explore the perceptions and acceptability of micro-sampling in children and young people.

Methods

Confidential: For Review Only

Online Patient and Public Involvement was obtained by attending the Generation R Young Person's Advisory Group Liverpool, to explore the perceptions of at home micro-sampling. During the session, the rationale and use of micro-sampling was demonstrated. The participants provided informal feedback during the interactive session and completed a short online questionnaire.

Results

A total of 13 children and young people attended the event and they were aged 10-17 years, of these 10 responded to an online questionnaire. The general feedback on micro-sampling was positive with 8/10% of respondents indicating they would be willing to participate in at home micro-sampling studies. Furthermore, 10/10 respondents reported being willing to provide both biofluids on a monthly basis and 4/10 would be willing to provide dried urine samples weekly.

Conclusions

Children and young people are supportive of at home micro-sampling for research purposes and this offers the possibility of widening participation to research.

What is known about the subject?

- New born screening using dried blood spots have been used for decades and the use of dried blood spots is adults is growing in popularity.
- The use of dried blood spots in adults has been shown to be applicable for many questions such as adherence to treatment regime.
- There is evidence from adults that they prefer this method of sample collection due to its less invasive nature particularly when economic and time costs are accounted for.

What this study adds?

- The small group of children and young persons in this study were happy with the collection of dried blood spots This opens the possibility for more children and young persons to contribute to medical research whom may have been excluded previously and may ease longitudinal sampling.

How this study might affect research, practice and/or policy

Confidential: For Review Only

- This may make decisions regarding ethical approvals easier given that this work gives this population a voice.
- These findings may help accelerate patient recruitment and sampling to help resolve the gap in research between paediatric and adult diseases.

Background

The collection of biofluid samples from patients for basic science and clinical research typically requires a patient to attend an appointment with a medical practitioner, particularly in the case of providing blood samples[1,2]. Even in the case of a urine sample, a visit to a hospital or research facility is frequently required, incurring further costs and time commitments to families. Recently, there has been a growing interest in micro sampling such as dried blood and urine spots, collected at home and posted back to the research centre for analysis[2]. The use of dried biofluids is cheaper[1,3,4] and an array of studies show the samples are stable over a few days which encompass the necessary postal time[5]. In addition, as a dried biofluid, the biological risk associated with the liquid form is significantly reduced[2,6]. Since the Covid19 pandemic, home testing of nasal/throat swabs have become common practice.

To date, there has been a growing number of studies evaluating home sampling and postage delivery spanning personalised nutrition[7], genome testing[8], disease monitoring and pre-clinical research[4,9,10]. In addition to the scientific questions at the heart of these studies, a number have looked into patients' perception and sample quality of micro-sampling. The overarching consensus is that participants are happy to provide blood, urine and saliva samples using micro sampling devices with <10% of patients preferring traditional clinical visits. The reasons for the latter being due to confusion over the sample collection, fear of pain with the finger prick blood devices or not feeling adequately trained in the sampling technique[11]. Furthermore, these studies report high levels of compliance with >90% of expected samples being taken and successfully arriving for analysis, even in the case of longitudinal sampling[3,12].

Confidential: For Review Only

These studies highlight the promise of at home micro-sampling to increase recruitment of patient populations that are not encompassed within a research-active hospital or university region and facilitate larger cohort recruitment with longitudinal sampling studies[4].

There is little data evaluating at home micro-sampling devices in paediatric cohorts, despite paediatric healthcare professionals being familiar with national newborn screening programmes. Using this technique in children and young people may also be dependent on the willingness of research ethics committees to grant ethical approval for at-home micro-sampling testing in a vulnerable population and thus there is a threat that children may be excluded from this research area. The aim of this work is to explore the perceptions of and willingness of children and young people to partake in research focused on at-home micro-sampling of biofluids.

Methods

Researchers (L.O (Female), A.J.C (Male)) applied to attend one of the GenR YPAG Liverpool meetings. The Liverpool YPAG was set up in 2006 and is open to young people between the ages of 8-23 years. The group currently has 28 members aged between 10-22 years old. Members either have experience of taking part in health research, have experience of living with a condition or disability, or have a general interest in learning about medicine/research/science. There were no inclusion or exclusion requirements for the study other than being a member of GenR YPAG group, any member was free to participate.

Prior to the workshop, meetings were held with the group facilitator (J.A) to plan the session as part of a regular GenR YPAG meeting and to support the purpose of the activity including expected outputs. The dissemination of a sample of the pack were also arranged. It was decided to send packs to a small group of members who would demonstrate to the rest of the group during the meeting. In total 6 mock sampling packs were sent to YPAG members to facilitate a live demonstration. In the mock sampling pack, volumetric absorptive micro-sampling (VAMS, Neoteryx, CA, U.S.A) devices were

https://mc.manuscriptcentral.com/bmjpo

Confidential: For Review Only

provided with 2 aliquots of food colouring to mimic blood and urine. Two aliquots of water were also provided to allow a demonstration of how to recover the dried simulated biofluids.

The online meeting was conducted over 45 minutes using Zoom video conferencing software (Zoom video communications, Ca, U.S.A), the session was not recorded. Researchers (L.O, J.A) presented current recruitment and sample collection practices and the importance of these for understanding inflammatory renal disease. The session subsequently involved a short video and a live interactive demonstration (A.J.C) to illustrate the sample collection process using the mock sampling kits. Mock samples (VAMS and food colouring) were left to dry for 20 minutes during the discussion phase prior to illustrating how to recover the dried sample using the provided water.

During the presentation a short video was shown demonstrating the use of a finger prick device for the generation of a capillary whole blood sample and its collection with a VAMS device (Figure 1). The group then discussed with the whole group for 35 minutes the positive and negative points to sample collection and finally a multiple-choice questionnaire (SurveyMonkey Audience www.surveymonkey.com/mp/audience) (Supplementary Information 1) was circulated to assess their perception and willingness for children and young people to partake in studies using micro-sampling devices. During the meeting written minutes were recorded by 2 individuals (L.O and J.M). The responses to the multiple-choice questionnaire was tallied and graphs produced (A.J.C) using GraphPad Prism Version 8.1.1 (GraphPad Software, CA, USA). The trends in perception were then garnered from this compiled data.

Figure 1: VAMS devices for the collection dried blood spots generated from 30 µL of capillary blood

Patient/public involvement

This work collected the views and perceptions of children and young persons from the public to inform and improve future at home dried biofluid micro-sampling clinical research studies.

For Review Only

Results

Participants

In total, 13 young people (46% of members, no parents present) attended the YPAG meeting via Zoom from home in April 2022. Only 1 young person of 6 received the mock pack and was able to attend due to educational and personal commitments. They confirmed that the mock sampling pack was able to fit through their letter box and had been received intact. Due to the small group data saturation was not reached.

Findings from informal discussion

When asked for first impressions on micro-sampling, the feedback was positive with comments on the method being "quick", "efficient", "simple", "helpful", "fast", "great" and "easy" being made. The participants were asked for any advantages of using these micro-sampling collection kits which followed a similar trend with responses of "Don't need a lot of volume of urine/blood", "simple to use and also fewer travelling costs" and "only takes ten minutes rather than a day to go to hospital".

Conversely, a number of concerns were raised which can either be readily resolved with added information or require additional work prior to rolling out micro-sampling (Table 1). The participant doing the hands-on demonstration mentioned that initially the procedure seemed "complicated" but ended up being "easy and simple to operate". This raised a further question "will there be an instruction guide or video?", the initial idea behind this form of sample collection was to provide a manual with a step by step process. However, the group suggested using a QR code with an embedded link to a guidance video or online manual.

An issue was raised about whether the results from these samples are comparable to standard onsite venous blood and urine sample collection. The issue of still having to collect a urine sample in a container prior to taking the microsample was also raised highlighting the need to investigate an appropriate container in the future. However, it was emphasised that collecting a urine sample at

https://mc.manuscriptcentral.com/bmjpo

Confidential: For Review Only

home was more convenient and more private compared to a hospital visit. The supply of finger prick devices in the packs to be shipped out was also questioned and it was confirmed that plans would be to include these with spares in case one did not work or multiple pricks were required. The final concern raised was the environmental impact of such testing, demonstrating the environmental concern of patients alongside medical concerns.

Table 1: Concerns raised by the group and the impact they may have on future studies with potential solutions discussed

**Concerns raised**	**Potential issues this would have**	**Possible solutions**
Complexity of sample collection/requirement for instruction	Poor quality sample<br>Unnecessary pain if blood taken incorrectly<br>Stress of participant	Develop manuals and videos in accessible language<br>Use of further PPI events to evaluate instructional manuals and videos.<br>Incorporation of QR codes to link to videos and manuals
Lack of data on how micro-sampling compared to traditional techniques	Lower quality data being collected<br>Not enough samples<br>Sample degradation	Prior to each study a method development phase is required to optimise sample recovery, limits of detection, sample stability.
Urine collection in a separate container	Inconvenient<br>Large volume to dispose of without spilling	Focus group highlighted that this is still preferable to in person sampling in clinic. However, it's something to

Confidential: For Review Only

		consider for researchers to the process as easy as possible.
Will the kits posted home contain the finger prick devices?	It could limit the recruitment of patients if they think they need to provide materials for sample collection	Full description of the process and kit contents in information packs for participants.<br>Ensuring adequate information in manuals and instructional videos.
Environmental impact of the micro-sampling kits	If perceived to be greater than traditional approaches it may deter participants.<br>It's important that clinical researchers consider the impact upon global pollution burden as this contributes to poor health outcomes.	At home sample collection reduces the need for transport to a research site.<br>Less plastics are required compared to traditional methods.<br>Researchers should assess a number of micro-sampling devices to minimise the use of single use plastics.

In terms of the ages that the group thought would be appropriate for micro-sampling, there was no obvious age cut-off. They suggested that it would need to be done on a case by case basis and felt that if young people under 16 years old were able to understand and consent then they should be allowed to participate and that some children and young people may actually prefer this method of sampling compared to traditional venous sample collection.

Confidential: For Review Only

Findings from questionnaire

The YPAG were asked to answer a multiple choice questionnaire (Supplementary information and Supplementary Table 1) to help evaluate their perceptions. Initial questions revolved around the use of micro-sampling devices for clinical research in paediatric cohorts. In total, 10/13 participants (77%) provided feedback to the questionnaire and of those, 9/10 felt that micro-sampling was preferential compared to having to visit the GP or a hospital site for blood drawing and 1/10 respondent was unsure. Furthermore, 8/10 of respondents thought that this approach would increase the likelihood of patients wishing to participate in clinical research while 2/10 were unsure.

A key question with this type of micro-sampling in this population is to understand which biofluids they feel comfortable with providing and this may provide challenging for ethical committees to make an informed decision on their behalf. Perhaps surprisingly, a great proportion were willing to provide a blood sample (9/10) compared to a urine sample (8/10), despite the use of a finger prick test to obtain the capillary blood sample. In the questions regarding both the perception of the micro-sampling devices and the biofluids, between 1/10 and 2/10 of respondents replied with 'unsure'.

Finally, the frequency of sampling is significant for the recruitment of patients over a defined time frame with periodic sampling providing a great opportunity for longitudinal data collection as the disease changes over time and treatment takes effect. Perhaps unsurprisingly, there was a trend for blood to be collected on a less regular basis, potentially due to apprehension regarding the finger prick testing. The frequency of urine testing was less clear cut with 4/10 suggesting that weekly collection or monthly collection would be acceptable (Figure 2).

Figure 2: The Liverpool YPAG opinions on the hypothetical acceptable frequency of micro-sampling depending upon sample type.

Discussion

Initial discussion

Confidential: For Review Only

The initial positive responses to the use of at home micro-sampling and return by mail reflect the findings in adult cohorts about the use of such devices that they would be quicker and easier to use and cut down on time and economic costs associated with a hospital or GP visit[12].

Importantly, a number of potential weaknesses were also highlighted by the group. The issues raised over the complexity and level of instruction for the at home sampling is a vital aspect of improving inclusivity enabling children, young people and parents to make informed choices and safely and reliably carry out the sample collection procedure. In order to be as inclusive as possible with this style of sampling, there is a clear need for providing instructions accessible for all to ensure safe and reliable at home micro-sample collection. A further PPI event would be beneficial to evaluate the manual or videos in order to optimise these for this audience. These issues are also reflected in adult cohorts where a number of participants requested further guidance for micro-sampling and that when it was made available participation rates were increased[3,11].

Concerns regarding quality of data that can be ascertained from micro-sampling compared to more traditional approaches is an understandable concern as patients may be asked to provide samples more frequently than typical during their clinical follow-up so it is important that researchers can reassure patients that the samples they are providing are being put to good use. In an array of studies in adult populations >90% of returned samples were of adequate volume and quality for research purposes, suggesting that this approach would be appropriate[3,11-13]. However, validation and stability for the specific analytes of interest will be required for all studies using this form of sample collection, particularly given that reference ranges are typically provided for serum or plasma rather than whole blood[11,14]. Once this has been validated, it would possibly be useful to include this information and additional information of previously published studies to show the applicability of the sampling technique to potential patients interested in providing dried biofluid samples.

The group highlighted a concern of the environmental impact of the plastics usage with this micro-sampling technique. Investigators will need to consider the type of plastic and that by removing the

https://mc.manuscriptcentral.com/bmjpo

Confidential: For Review Only

requirement to travel to a research centre and the reduced sample processing in the lab[1,3] may balance the environmental impact compared to conventional sampling. Furthermore, the suggestion of QR codes for access to videos and manuals could reduce paper work and the weight of the packaging however this may skew against individuals in lower socio-economic backgrounds without access to take advantage of this.

PPI Questionnaire

The reported willingness to participate in this style of study is much higher than the response rate observed in an adult epidemiological cohort and may reflect that this was a small group or the difference between a theoretical study and one that is actually taking place[13]. This is key as this population is already under represented in the literature potentially due to the low numbers of specialist paediatric research facilities, spare biofluid volume for clinical research or reticence to provide ethical consent for clinical research on this population. All of which combine to limit the amount of research on paediatric diseases potentially leaving this population behind in terms of mechanistic insights, discovery of potential drug targets and the development of new drugs.

The slight preference for blood sampling over urine is particularly interesting because urine is often stated as a preferred biofluid for research due to it being less invasive and more plentiful. It is possible that this is down to having to provide a sample into a large pot and then taking a sample or embarrassment around providing a urine sample. In addition, the disposal of a container full of urine once the sample has been taken may also put participants off.

The YPAG group responded that they would be open to longitudinal collection of biofluids, though there was no consensus regarding the frequency of sample collection. The results suggested that aiming monthly sampling may be acceptable however further work using specific cohorts may be required.

Study Limitations

Confidential: For Review Only

While the findings from this work are promising for at home micro-sampling in young persons and children there are number of important limitations to consider. This study only used a very small number of individuals (n=13) spanning a relatively large age range, additionally only 10 responded to the questionnaire. These individuals also have an interest in medical research and members of the Liverpool GenR younger persons advisory group. As such these individuals are potentially more inclined to find this approach favourable. It is also very difficult to simulate the experience of a finger prick test only a video was used to simulate this and its probable that individuals may change their perceptions of this approach following their first experience. However, this effect may be less evident for the use of dried urine spots. This work was also complicated by the fact only one participant has received the at home demo kit to evaluate for the session. This work offers some insight into the views and thoughts on this topic but larger follow up studies before roll out or with the roll out of at home micro-sampling will be vital to evaluate the views of young adults and children on this process.

Conclusions

While the group size is small and there are biases due to the intellect and interest in clinical research held by members possibly skewing the findings, we are able to report that children and young people may be willing to provide samples in this manner. We also gained insight on the frequency and additional concerns that need to be addressed for longitudinal research studies aimed at elucidating mechanistic insights into paediatric diseases. These findings may help applications to ethics boards for this style of research now that there are limited data on the views of this age group.

Acknowledgements

We would like to thank the Liverpool GenR YPAG for allowing us to present our proposal and for their critical and thoughtful feedback to this work.

References

Confidential: For Review Only

1 Locatelli M, Tartaglia A, D'Ambrosio F, *et al.* Biofluid sampler: A new gateway for mail-in-analysis of whole blood samples. *Journal of Chromatography B* 2020;**1143**:122055. doi:10.1016/J.JCHROMB.2020.122055

2 Resano M, Belarra MA, García-Ruiz E, *et al.* Dried matrix spots and clinical elemental analysis. Current status, difficulties, and opportunities. *TrAC Trends in Analytical Chemistry* 2018;**99**:75–87. doi:10.1016/J.TRAC.2017.12.004

3 Roberts SC, Seav SM, McDade TW, *et al.* Self-collected dried blood spots as a tool for measuring ovarian reserve in young female cancer survivors. *Human Reproduction* 2016;**31**:1570–8. doi:10.1093/HUMREP/DEW114

4 Parker SP, Cubitt WD. The use of the dried blood spot sample in epidemiological studies. *J Clin Pathol* 1999;**52**:633–9.

5 Nakajima D, Ohara O, Kawashima Y. Toward proteome-wide exploration of proteins in dried blood spots using liquid chromatography-coupled mass spectrometry. *Proteomics* 2021;**21**:2100019. doi:10.1002/PMIC.202100019

6 Schmidt J, Lindemann V, Olsen M, *et al.* Dried urine spots as sampling technique for multi-mycotoxin analysis in human urine. *Mycotoxin Res* 2021;**37**:129–40. doi:10.1007/S12550-021-00423-1/TABLES/3

7 Hoeller U, Baur M, Roos FF, *et al.* Application of dried blood spots to determine vitamin D status in a large nutritional study with unsupervised sampling: the Food4Me project. *British Journal of Nutrition* 2016;**115**:202–11. doi:10.1017/S0007114515004298

8 Agrawal P, Katragadda S, Hariharan AK, *et al.* Validation of whole genome sequencing from dried blood spots. *BMC Med Genomics* 2021;**14**:1–11. doi:10.1186/S12920-021-00951-W/FIGURES/2

9 Solomon SS, Solomon S, Rodriguez II, *et al.* Dried blood spots (DBS): A valuable tool for HIV surveillance in developing/tropical countries. *Int J STD AIDS* 2002;**13**:25–8. doi:10.1258/0956462021924578

10 Chace DH, Millington DS, Terada N, *et al.* Rapid diagnosis of phenylketonuria by quantitative analysis for phenylalanine and tyrosine in neonatal blood spots by tandem mass spectrometry. *Clin Chem* 1993;**39**:66–71. doi:10.1093/CLINCHEM/39.1.66

11 Williams SR, McDade TW. The Use of Dried Blood Spot Sampling in the National Social Life, Health, and Aging Project. *The Journals of Gerontology: Series B* 2009;**64B**:i131–6. doi:10.1093/GERONB/GBN022

12 Spielberg F, Critchlow C, Vittinghoff E, *et al.* Home collection for frequent HIV testing: acceptability of oral fluids, dried blood spots and telephone results. *Lippincott Williams & Wilkins AIDS* 2000;**14**:1819–28.http://journals.lww.com/aidsonline (accessed 31 Mar 2022).

13 Sakhi AK, Bastani NE, Ellingjord-Dale M, *et al.* Feasibility of self-sampled dried blood spot and saliva samples sent by mail in a population-based study. *BMC Cancer* 2015;**15**:1–9. doi:10.1186/S12885-015-1275-0/TABLES/3

14 McClendon-Weary B, Putnick DL, Robinson S, *et al.* Little to Give, Much to Gain—What Can You Do With a Dried Blood Spot? *Curr Environ Health Rep* 2020;**7**:211–21. doi:10.1007/S40572-020-00289-Y/FIGURES/1

Confidential: For Review Only

Supplementary Information

How are micro-sampling and home testing kits perceived by children and young people?

Andrew J Chetwynd[1,2], Julien Marro[1], Laura Whitty[1], Jenny Ainsworth[3], Jennifer Preston[1], Alan Salama[4], Louise Oni[1,5]

[1] Department of Women's and Children's Health, Institute of Life Course and Medical Sciences, University of Liverpool, Liverpool L12 2AP, UK

[2] Centre for Proteome Research, Institute of Systems, Molecular and Integrative Biology, University of Liverpool, Liverpool, U.K.

[3]NIHR Alder Hey CRF, Institute in the Park, Alder Hey Children's NHS Foundation Trust, Eaton Rd, Liverpool, L12 2AP

[4] Department of Renal Medicine, University College London, London, United Kingdom

[5] Department of Paediatric Nephrology, Alder Hey Children's NHS Foundation Trust Hospital, Liverpool L12 2AP, UK

Page 2: Questionnaire used during PPIE event

Page 3: Compiled responses to questionnaire in table format

Confidential: For Review Only

Questionnaire for PPIE event held on 7th April 2022

Do you think young people with inflammatory diseases would prefer this method compared to visiting Doctors or hospital?

Yes/No/Unsure

Do you think young people with inflammatory diseases would be more likely to participate in a clinical research study using this method?

Yes/No/Unsure

Do you think young people with inflammatory diseases would be willing to use these devices to provide urine samples?

Yes/No//Unsure

How frequently do you think young people with inflammatory diseases would be happy to provide a urine sample?
Weekly/every 2 weeks/monthly/every 3 months/less often/Unsure

Do you think young people with inflammatory diseases would be willing to use these devices to provide blood samples?

Yes/No/Unsure

How frequently do you think young people with inflammatory diseases would be happy to provide a blood sample?
Weekly/every 2 weeks/monthly/every 3 months/less often/Unsure

Did the pack fit through your letter box?

Yes/No/Unsure

Confidential: For Review Only

Table 1: Compiled results of questionnaire

Question	Yes	No	Unsure	Once a week	Every 2 weeks	Once a month	Once every 3 months	Less often
1	9	0	1	N/A	N/A	N/A	N/A	N/A
2	8	0	2	N/A	N/A	N/A	N/A	N/A
3	8	0	2	N/A	N/A	N/A	N/A	N/A
4	N/A	N/A	0	4	2	4	0	0
5	9	0	1	N/A	N/A	N/A	N/A	N/A
6	N/A	N/A	0	2	3	5	0	0
7*	1	0	0	N/A	N/A	N/A	N/A	N/A

*n=1 as only one participant who was able to attend had received a pack, another participant who had been shipped a pack had yet to receive it.

Confidential: For Review Only

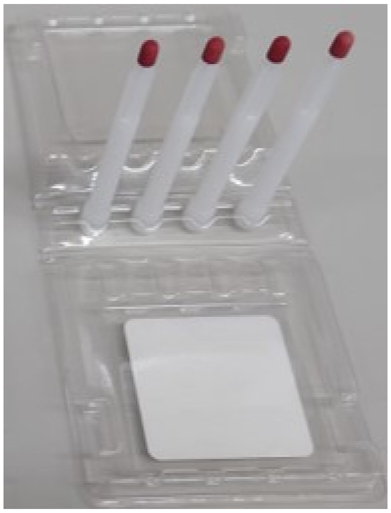

16x21mm (300 x 300 DPI)

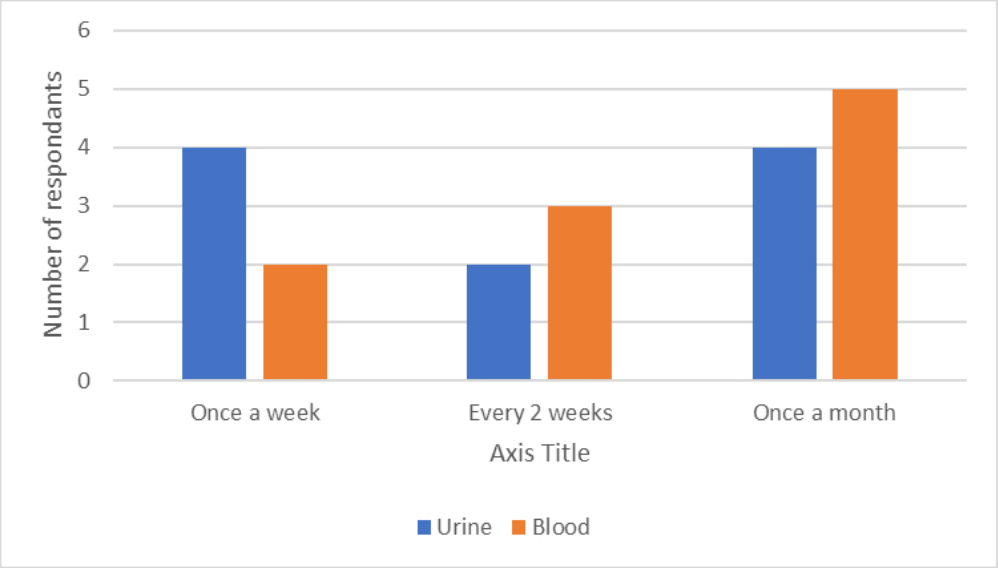

63x36mm (300 x 300 DPI)
